# In Vivo Effects of a GHR Synthesis Inhibitor During Prolonged Treatment in Dogs

**DOI:** 10.3390/ph17101381

**Published:** 2024-10-16

**Authors:** Elpetra P. M. Timmermans, Joëlle Blankevoort, Guy C. M. Grinwis, Sietske J. Mesu, Ronette Gehring, Patric J. D. Delhanty, Peter E. M. Maas, Ger J. Strous, Jan A. Mol

**Affiliations:** 1 Department Clinical Sciences, Faculty of Veterinary Sciences, Utrecht University, 3584 CM Utrecht, The Netherlandsj.a.mol@uu.nl (J.A.M.); 2 Department of Biomolecular Health Sciences, Pathology Division, Faculty of Veterinary Medicine, Utrecht University, 3584 CM Utrecht, The Netherlands; g.c.m.grinwis@uu.nl; 3 Department Population Health Sciences, Institute for Risk assessment Sciences (IRAS), 3584 CM Utrecht, The Netherlands; s.j.mesu@uu.nl (S.J.M.); r.gehring@uu.nl (R.G.); 4 Department Internal Medicine, Endocrinology, Erasmus Medical Centre, 3015 GD Rotterdam, The Netherlands; p.delhanty@erasmusmc.nl; 5 Specs Compound Handling B.V., 2712 PB Zoetermeer, The Netherlands; peter.maas@specs.net; 6 Center for Molecular Medicine, Cell Biology, University Medical Center Utrecht, 3508 AB Utrecht, The Netherlands; strou046@planet.nl

**Keywords:** small molecule, dog, IGF-1, GH, toxicity, ghrelin

## Abstract

**Background:** The activation of the growth hormone receptor (GHR) is a major determinant of body growth. Defective GHR signaling, as seen in human Laron dwarfism, resulted in low plasma IGF-1 concentrations and limited growth, but also marked absence in the development of breast cancer and type 2 diabetes. In vitro, we identified a small molecule (C#1) that inhibits the translation of GHR mRNA to receptor protein. **Methods:** Before its application in humans as a potential anticancer drug, C#1 was tested in animals to evaluate whether it could be administered to achieve a plasma concentration in vivo that inhibits cell proliferation in vitro without causing unwanted toxicity. To evaluate the efficacy and toxicity of C#1, a group of six intact female Beagle dogs was treated daily each morning for 90 days with an oral solution of C#1 in Soiae oleum emulgatum at a dose of 0.1 mg/kg body weight. During treatment, dogs were closely monitored clinically, and blood samples were taken to measure plasma C#1 concentrations, complete blood counts (CBC), clinical chemistry, and endocrinology. At the end of the treatment, dogs were euthanized for gross and histopathological analysis. An additional group of six female Beagle dogs was included for statistical reasons and only evaluated for efficacy during treatment for 30 days. **Results:** Daily administration of C#1 resulted in a constant mean plasma concentration of approximately 50 nmol/L. In both groups, two out of six dogs developed decreased appetite and food refusal after 4–5 weeks, and occasionally diarrhea. No significant effects in CBC or routine clinical chemistry were seen. Plasma IGF-1 concentrations, used as biomarkers for defective GHR signaling, significantly decreased by 31% over time. As plasma growth hormone (GH) concentrations decreased by 51% as well, no proof of GHR dysfunction could be established. The measured 43% decrease in plasma acylated/non-acylated ghrelin ratios will also lower plasma GH concentrations by reducing activation of the GH secretagogue receptor (GHSR). C#1 did not directly inhibit the GHSR in vivo*,* as shown in vitro. There were no significant effects on glucose, lipid, or folate/homocysteine metabolism. **Conclusions:** It is concluded that with daily dosing of 0.1 mg C#1/kg body weight, the induction of toxic effects prevented further increases in dosage. Due to the concomitant decrease in both IGF-1 and GH, in vivo inhibition of GHR could not be confirmed. Since the concept of specific inhibition of GHR synthesis by small molecules remains a promising strategy, searching for compounds similar to C#1 with lower toxicity should be worthwhile.

## 1. Introduction

The growth hormone receptor (GHR) is widely expressed throughout the body and regulates tissue growth and differentiation [1]. Once activated, it is not surprising that the GHR may also play an important role in regulating cancer and chronic diseases [2]. In breast cancer, the GHR promotes progression via the MAPK pathway [3]. Remarkably, patients with defective GHR signaling caused by an inactivating mutation of the GHR, as found in Laron dwarfism, do not develop cancer [4,5]. GHR signals intracellularly by JAK2/STAT, JAK2/PI3K, or SRC/MAPK pathways [6].

For activation of GHR, the growth hormone (GH) ligand must bind to a preformed intracellular GHR dimer [2]. Most of the plasma GH is derived from the pituitary gland and is secreted in a pulsatile fashion. However, during pregnancy, the major GHR ligand in plasma is the placental variant form GH2. Next to pituitary GH, locally produced GH may also activate the GHR. In normal human breast epithelium, GH is secreted after progesterone stimulation and results in the proliferation of stem/progenitor cells [7].

Local progestin-induced production of GH in the mammary gland was first shown in dogs and is mediated by the progesterone receptor-A variant [8]. Expression of the gene encoding GH was next shown in mammary gland carcinomas of dogs and humans [9]. Also, GHR is expressed not only in epithelial cells but also activates fibroblasts in the tumor stroma of normal and neoplastic mammary tissue in dogs [8]. Inhibition of the local GH/GHR activity could be an attractive target to inhibit unwanted proliferation of mammary cells in a variety of species [8]. In mammary tumor cells, GH and GHR may be co-expressed, resulting in the autocrine intracellular activation of the GH/GHR system [2,6], which is inaccessible for drugs that only compete for the GH/GHR interaction at the cell surface [10]. This was the impetus to search for small molecule inhibitors to regulate the GH/GHR system intracellularly [11]. In that study, five lead compounds, including C#1 (BM001), reduced the in vitro cell proliferation of the MDA-MM-231 triple-negative breast cancer cell line and the Colo-205 cell line at dose rates between 10–50 nM. The most promising GHR-inhibiting compound, C#1, showed a 6-fold decrease in tumor growth in mice transplanted with the triple-negative mammary breast cancer cell line MDA-MM-231, at a dose of 1 mg/kg, three times weekly [11]. In the present study, C#1 was further tested for its effectiveness in growth hormone receptor (GHR) inhibition, plasma IGF-1 reduction, biological activity, and toxicity in Beagle dogs.

## 2. Results

### 2.1. Physical and Metabolic Properties of C#1

C#1 was found to be very poorly soluble in water, 10 mM PBS (<2 μg/mL), or ethanol, while it was easily soluble up to 50 mg/mL in DMSO. For in vitro experiments, a stock solution of C#1 in DMSO was chosen. For in vivo administration, a solution in Soiae oleum emulgatum of 5 mg/mL was used. After adding C#1 to human, mouse, or dog plasma, no decrease in concentration was seen for 240 min. With equilibrium dialysis, it was determined that >96% of the C#1 was bound to plasma proteins. Incubation with liver microsomes (human, mouse, dog) showed high stability of C#1, with corresponding half-lives of >100 min and no inhibition at 20 μM of human CYP450 isoenzymes 1A2, 2B6, 2C8, 2C9, and 2C19, and high IC50 values of approximately 20 μM for inhibition of CYP3A4 and 2D6 activity (Table S1 and Figure S1).

### 2.2. Pilot Administration of C#1

In the initial studies in mice with human breast cancer cell line xenografts, a maximum intraperitoneal dose of 5 mg/kg body weight was given 3 times a week for 21 days without external signs of toxicity [2,11]. However, oral administration is preferred for longer use. A single dose of 5, 10, and 15 mg C#1/kg body weight in corn oil in SCID mice resulted in a plasma peak concentration of 0.92, 1.8, and 2.9 µM, 4 to 8 h after oral administration*,* and an estimated mean plasma half-life of 40 h. Subsequently, SCID mice (*n* = 6) were given oral C#1 in corn oil 3 times a week at a concentration of 5, 10, and 15 mg/kg body weight for 18 days. The lowest concentration was well tolerated, but the highest dose of 15 mg/mL resulted in dull behavior and loss of animals after 8–10 days.

Because the bioavailability after oral administration was high in mice, it was decided to give a dog a single dose of 1 mg/kg. This resulted in a plasma level of 538 nM after 8 h and an estimated half-life of 48 h. Three days after oral administration, the test dog became seriously ill and reached a humane endpoint. Pathological examination could not establish a link with the administration of C#1. Nevertheless, it was decided to give a subsequent test dog a dose of 0.5 mg/kg, resulting in a plasma level of 167 nM at 4–8 h after administration and again a half-life of 48 h. In this dog, no side effects were noted.

### 2.3. Efficacy and Toxicity Trial During Prolonged Treatment of Female Beagle Dogs

Based on the plasma levels and half-lives measured in the pilot experiment, it was decided to administer C#1 daily in the morning at a dose of 0.1 mg/kg. The first group (*n* = 6) was given C#1 daily for 90 days. On day 36, one of the six dogs stopped eating (anorexia grade 1/2), which led to general malaise. However, the animal remained alert and showed no vomiting or diarrhea, which did not result in a humane endpoint. It was then decided to discontinue the administration of C#1 in this animal. No abnormalities were found on physical examination, blood examination, and abdominal ultrasound. The animal was treated with an antiemetic (maropitant, Cerenia^®^) to improve appetite. After consultation with veterinary specialists and laboratory animal experts, it was decided to resume administration of C#1 on day 50 after sufficient recovery. On day 60, food refusal recurred in the same animal and also in a second animal in the study. It was then decided that both animals should be removed from the experiment permanently. After recovery and approval from the animal welfare body, on day 75, only the vehicle (Soiae oleum emulgatum) was administered to the two dogs that were removed from the experiment to confirm that the cessation of eating was due to compound C#1 and not to the daily administration of 0.25 mL of Soiae oleum emulgatum in which the substance was dissolved. Inappetence did not occur after the administration of the vehicle only for 25 days in either dog. Due to the adverse effects in these two animals, the dose in the second group was not increased, and animals were treated with the same dose of C#1 for 30 days. On day 23, again, two animals were found to have reduced appetite. Clinically, however, no abnormalities were found on physical examination. One animal showed further deterioration (diarrhea with blood loss and vomiting) on day 25. It was decided to remove this animal from the study on day 26 and to take a blood sample for clinical chemistry on that day. The animal quickly recovered.

### 2.4. Results After 5 Weeks of C#1 Administration

**Figure 1:**
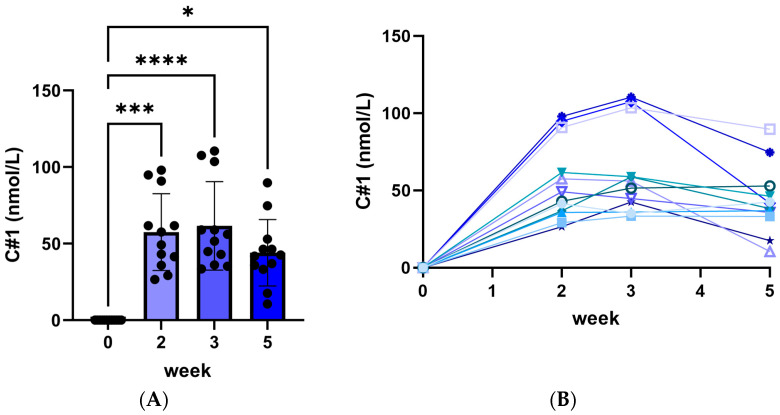
Plasma C#1 concentrations after daily administration of 0.1 mg C#1/kg body weight to female Beagle dogs. (**A**) median C#1 concentrations for all 12 dogs with significance * *p* < 0.05, *** *p* < 0.005 and **** *p* < 0.001. (**B**) each dog’s individual C#1 concentration per week. All lines stand for the individual plasma C#1 concentration for each dog during the experiment.

The following results are from the first 5 weeks of all animals in both cohorts (*n* = 12). During the first 5 weeks, no significant change in body weight was measured (mean: 12.0 ± 0.6 kg body weight). Plasma C#1 concentrations averaged 55.4 ± 7.4, 61.6 ± 8.3, and 43.4 ± 6.3 nmol/L at 2, 3 and 5 weeks of treatment, respectively (Figure 1, left panel). Individual values (Figure 1, right panel) showed a significant variation in plasma C#1 levels.

Routine clinical chemistry was measured in plasma (Table 1). No major changes were found. At week two, a significant increase in plasma urea was found, which then returned to normal at week five, next to a simultaneous transient increase in plasma folic acid and trypsin-like immunoreactivity (TLI) and a decrease in vitamin B12 concentrations. At week five, a significant reduction in total bilirubin levels was found.

Complete blood count (CBC) showed no significant changes in blood counts (Table 2).

**Figure 2:**
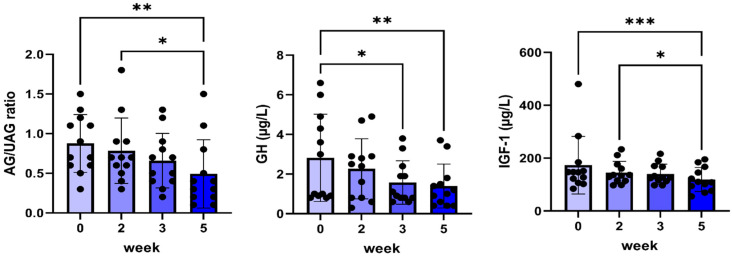
Plasma ratio of acylated/unacylated ghrelin (AG/UAG), GH, and IGF-1 before and during 5 weeks of daily oral treatment of female Beagle dogs with 0.1 mg/kg C#1. Significance: * *p* < 0.05, ** *p* < 0.01, *** *p* < 0.005.

Plasma IGF-1 concentrations were used as a surrogate marker for the inhibition of GHR activity. A significant decrease in plasma IGF-1 concentrations was found after five weeks of treatment (Figure 2). This decrease was associated with a reduction in plasma GH concentrations at weeks three and five as well as decreased plasma ghrelin concentrations, depicted as the ratio between acylated and unacylated ghrelin concentrations (Figure 2).

**Figure 3:**
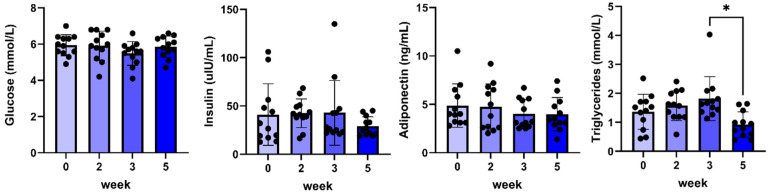
Plasma glucose, insulin, adiponectin, and triglyceride concentrations before and during 5 weeks of daily oral treatment of female Beagle dogs with 0.1 mg/kg C#1. Significance: * *p* < 0.05.

No changes were found in plasma glucose, insulin, and adiponectin concentrations (Figure 3), whereas only a small but significant decrease in plasma triglycerides was found between weeks three and five.

**Figure 4:**
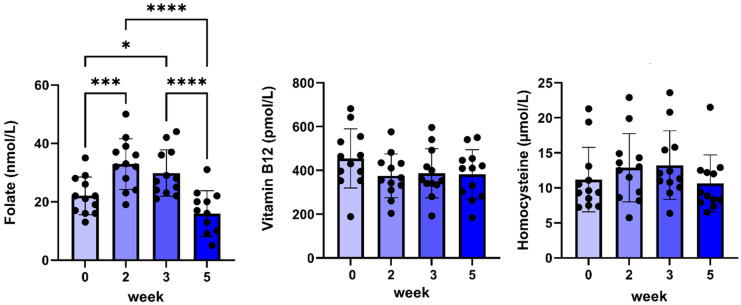
Plasma folate, vitamin B12, and homocysteine concentrations before and during 5 weeks of daily oral treatment of female Beagle dogs with 0.1 mg/kg C#1. Significance: * *p* < 0.05, *** *p* < 0.005, **** *p* < 0.001.

Finally, during weeks two and three, an increased plasma folate concentration was found, which normalized at week five, whereas no changes in plasma vitamin B12 or homocysteine concentrations were found (Figure 4).

### 2.5. Results from 90 Days of C#1 Administration

Treatment longer than 30 days resulted in grade 1/2 toxicity in two out of six dogs, characterized by food refusal, increased abdominal tension, and sometimes diarrhea associated with a 10% loss of body weight. In the other four dogs, no changes in body weights were found. Food refusal was associated with the highest C#1 plasma concentrations. It was decided to remove the two dogs with toxicity from the study. Treatment of these dogs with the vehicle of Soiae oleum emulgatum did not result in toxicity, indicating that C#1, but not the vehicle, was causative.

The plasma concentrations of alkaline phosphatase and the transaminase ALAT decreased further during the study. Plasma thyroxine concentrations increased gradually during the study from 17.5 ± 3.6 nmol/L to 31.5 ± 12.2, while remaining within reference values. At the end of the study, urinary corticoid/creatinine ratios markedly increased, indicating enhanced stress levels. No major differences from week five onward were noticed in the other parameters measured.

**Figure 5:**
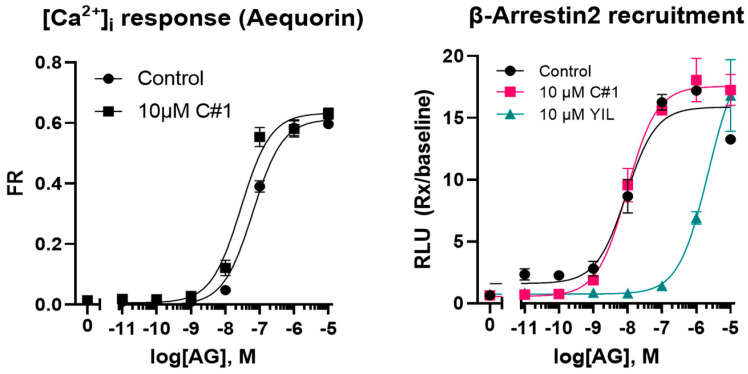
Effect of C#1 on acylated ghrelin (AG) mediated calcium influx or ß-arrestin recruitment. The GHSR antagonist YIL781 was used as positive control.

Although plasma IGF-1 concentrations, used as biomarkers for the effect of GHR inhibition, declined during treatment, the current data cannot confirm the proposed mode of action of GHR inhibition as the plasma GH concentrations also declined. Because of the food refusal, the similarity of the C#1 structure and inhibition of dihydrofolate reductase (DHFR) activity by C#1 at higher concentrations (see Discussion Section), the in vitro inhibitory activity of GHSR activity by C#1 was investigated. However, there were no indications that C#1 had any GHSR inhibitory activity (Figure 5).

At the end of the 90-day treatment, dogs were euthanized and investigated for macroscopic and microscopic changes in various tissues.

### 2.6. Pathology

No microscopic lesions were found in oral or nasal mucosa or the colon. No pathology or slight superficial gastritis was found in the stomach. However, mild lymphoplasmacytic enteritis was found in the duodenum, jejunum, and ileum (Table 3).

In the pituitary gland, predominantly acidophilic, possibly somatotropic, cells were seen. Adrenals often contained hyperplastic nodules without cortical atrophy.

## 3. Discussion

The growth hormone receptor (GHR) is crucial for transmitting signals from the growth hormone (GH) to the cells responsible for normal growth, development, and metabolism. Extensive studies have focused on how the GHR is activated and broken down [1]. GH stimulates the growth of almost all tissues, directly by activation of quiescent stem cells, or indirectly by stimulating the release of insulin-like growth factor-1 (IGF-1), resulting in both anabolic and catabolic actions of GH [12]. Aberrations in the GH signaling pathway have been connected to various medical conditions, including cancer [2]. Interestingly, a defect in the GHR, as seen in Laron syndrome, protects from cancer [4]. Therefore, inhibition of GHR activity may be effective in inhibiting tumor cell proliferation. However, current drugs, like the GHR antagonist Pegvisomant^®^, only inhibit GHR activity when expressed at the outer cell surface by competition with GH binding [6] and are used to mitigate the effects of acromegaly [13], whereas co-expression of GH and the GHR in cancer cells may result in intracellular autocrine activation that will not be inhibited by classic GHR antagonists [2]. This was the impetus to study small molecules that can inhibit GHR synthesis [11]. The most active compound (C#1) used in previous studies showed a strong decrease in tumor volume of mice transplanted with an aggressive human breast cancer cell line, MM231. The current study investigated the effects on general health parameters of repeated or prolonged administration of C#1 in mice and dogs.

Oral treatment is probably the most practical route of administration for extended periods of treatment and potential medical applications. As C#1 is hardly soluble in water, administration in oil was assessed in mice. A single oral dose of 5 mg/kg already resulted in high bioavailability, peak plasma concentrations around 1 µM, and a long plasma half-life in line with high plasma protein binding and limited biotransformation by liver microsomes. A dose-limiting toxicity was found at three-week oral dosages of 15 mg/kg. Due to the long plasma half-life, good oral bioavailability, and the in vitro IC50 of 10–30 nM [11], it was decided to use a daily dose of 0.1 mg C#1/kg bodyweight for 90 days for the toxicity study in dogs.

The effect of C#1 is attributed to a reduction in both the endo- and endocrine activities of the GH/IGF1 axis with the potential to inhibit GH-driven cancer growth [11], although the same compound appeared to have DHFR inhibitory effects in vitro as well [14] and acts as an antifolate. Therefore, the main question is whether C#1 is biologically active via GHR inhibition or an antifolate mechanism and to gain more information about its toxicity in vivo.

### 3.1. GHR Inhibition

**Figure 6:**
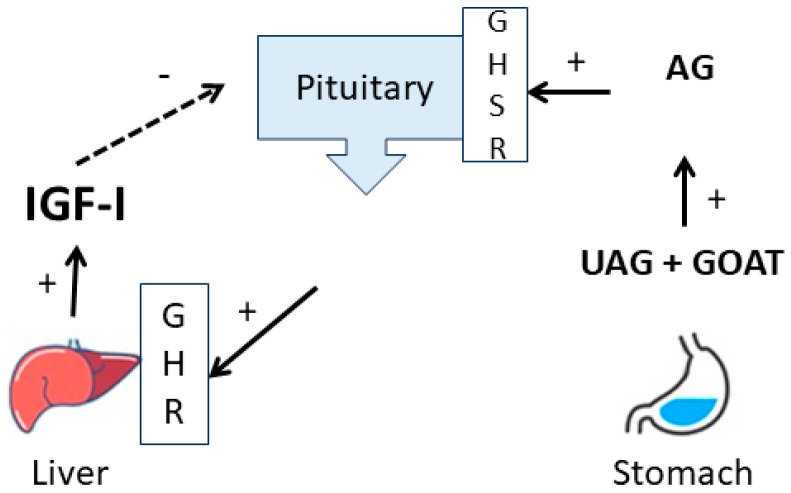
Schematic overview of the relation between ghrelin and the GH IGF-1 axis. Unacylated ghrelin (UAG) from the stomach is acylated by the Ghrelin O-Acyltransferase (GOAT) to acylated ghrelin (AG) and stimulates pituitary GH release.

Pituitary GH stimulates tissues to produce IGF-1 in plasma predominantly derived from the liver (Figure 6).

Blocking of GHR function would therefore result in decreased IGF-1 production and a concomitant rise of plasma GH due to the absence of negative feedback. But, although we measured a significant decrease in plasma IGF-1, no increase in plasma GH occurred. On the contrary, plasma GH concentrations also decreased. This was associated with a decrease in active acylated ghrelin concentrations. Similarities in the structure of C#1 GHSR inhibitors that also inhibit DHFR activity at higher concentrations [15], and the food refusal in some dogs, raised the question of whether C#1 would act as a ghrelin antagonist and thus GHSR inhibitor, which was not the case. A defect in GHR function is also associated with increased insulin sensitivity, higher adiponectin concentrations, and decreased triglyceride concentrations [16]. However, although a tendency of decreased plasma insulin concentrations with constant glucose levels and lower triglyceride concentrations at the end of the study was found, no changes in adiponectin concentrations were found, indicating that no major GHR defects occurred at the current dose and duration of treatment. Recently, decreased GHR expression by C#1 was confirmed in hNCC cell line experiments at a dose range of 25–50 nM [17], comparable to the concentrations achieved in the current trial. However, as the plasma binding of C#1 is high, the free biologically active concentration may be lower than in cell line experiments with 5% serum. It is concluded from our data that a GHR defect cannot be excluded, but other effects on GH release impede definitive conclusions.

In vitro experiments further showed that the possibility that C#1 acts as a GHSR antagonist is highly unlikely. It remains to be investigated whether C#1 can inhibit the ghrelin acylation by the Ghrelin O-Acyltransferase (GOAT) enzyme.

### 3.2. DHFR Inhibition

Concerning DHFR inhibition, plasma homocysteine may function as a good biomarker, as homocysteine concentrations will rise due to decreased tetrahydrofolate (THF) concentrations [18]. Because folate or vitamin B12 deficiency will also result in increased plasma homocysteine levels, this mechanism should be excluded as a cause of changed plasma homocysteine concentrations. No changes in vitamin B12 concentrations were found. However, plasma folate concentrations temporarily increased during C#1 treatment. Folate concentrations may rise due to small intestinal bacterial overgrowth or because of endocrine pancreas insufficiency. The latter was not the case, as plasma trypsin-like immunoreactivity (TLI) did not decrease during treatment. Whether plasma folate concentrations rise due to DHFR inhibition is questionable. Moreover, no significant increase in plasma homocysteine concentrations, which should be a clear indication of DHFR inhibition, was observed in our trial. The higher concentrations found in two dogs were already present before C#1 administration and, thus, unrelated to C#1. Antifolates, such as methotrexate (MTX), may also cause thrombocytopenia or hepatotoxicity, which were also not found in our study. It is therefore concluded that no clear indications of DHFR inhibition were found at the dose used. Antifolates like MTX may also function as Jak/Stat inhibitors [18]. The underlying mechanism is unclear but could align with the putative GHR inhibition and the subsequent decreased plasma IGF-1 concentrations. Although this mechanism cannot be ruled out, the decreased plasma GH concentrations may also be the cause of lower IGF-1. So again, direct inhibition by C#1 of the GHR-induced Jak/Stat signaling cannot be proven in the current trial.

### 3.3. Toxicity

Prolonged treatment of dogs with C#1 resulted in gastrointestinal toxicity and food refusal in two out of six dogs. This hampered further dose escalation in association with prolonged treatment. In these two dogs, mild signs of gastritis and enteritis were found at pathological evaluation at the end of the trial. This was because of C#1 administration cessation, already 5 weeks after the last dosage. Nevertheless, we assume a relation with the gastrointestinal problems that these dogs were facing. In the older dogs of the first cohort, a variety of neoplasms were found that were not clear at the health check at the start of the trial. These kinds of neoplasms are not uncommon in elderly dogs, but the C#1 treatment was not effective in preventing these neoplasms. As seen in the late and limited, but significant decrease in plasma IGF-1 concentrations, the effect could be improved by increasing the dose of C#1.

## 4. Materials and Methods

### 4.1. Animals

Healthy intact female Beagle dogs belonging to the kennel of the Department of Clinical Sciences were used. As the drug is primarily intended to treat dogs with mammary gland carcinoma, only elderly female dogs were included. All experimental details were approved by the animal welfare body Utrecht of Utrecht University, the Netherlands (registration AVD1080020186504).

A first cohort of 6 female Beagle dogs with a mean age of 10.9 ± 1.7 years and a mean body weight of 13.1 ± 1.8 kg was treated for 90 days with C#1. Dogs received 0.1 mg C#1/kg body weight orally every morning, followed by a spoon of Hill’s Restorative Care a/d^®^ food (Hill’s Pet Nutrition, Topeka, KS, USA). Blood samples were obtained by venipuncture before the start of the experiment (control sample), at weeks 2 and 3, and then every two weeks until the end of treatment. Animals were euthanized, a board-certified veterinary pathologist performed a comprehensive autopsy, and tissue samples of organs deemed relevant to the study were collected and fixed in 4% neutral buffered formalin for histopathological evaluation. A second cohort of 6 female Beagle dogs with a mean age of 4.6 ± 3.0 years and a mean body weight of 11.2 ± 1.4 kg were treated for 30 days following the same procedure and sampling protocol, though without the pathological evaluation, and were returned to the kennel afterward.

### 4.2. Materials

Compound C#1 was obtained from Specs Compound Handling (Zoetermeer, the Netherlands). For oral administration, C#1 was dissolved (using an ultrasonic bath) in Soiae oleum emulgatum (Bufa, IJsselstein, the Netherlands) to a concentration of 5 mg/mL at the pharmacy of the faculty of veterinary medicine, Utrecht University. The solution was filled out in dark glass vials and kept at 2–4 °C until use.

### 4.3. Assays

Routine clinical chemistry was performed at the University Veterinary Diagnostic Laboratory (UVDL). Blood parameters were measured using the Advia analyzer (Siemens Healthineers, The Hague, the Netherlands) and consisted of hemoglobin, hematocrit, reticulocytes, leukocytes, lymphocytes, monocytes, neutrophils, and thrombocytes. Clinical chemistry was measured using the AU480 chemistry analyzer (Beckman Coulter, Woerden, the Netherlands) and included urea, creatinine, glucose, alkaline phosphatase, ALAT, ASAT, bile acids, triglycerides, and total bilirubin. Plasma insulin, IGF-1, cortisol, progesterone, total thyroxine, homocysteine, folic acid, vitamin B12, and trypsin-like immunoreactivity (TLI) were measured using the Immulite 2000 system (Siemens). Plasma growth hormone (GH) was measured using an in-house assay for dogs [19]. Adiponectin, acylated ghrelin, and unacylated ghrelin were measured using a specific ELISA (Adiponectin BioVendor, Brno, Czech Republic, and both ghrelin kits from Bertin technologies, Aix en Provance, France). GHSR activity was measured using aequorin (calcium response) and beta-arrestin2 recruitment assays. The effects of pre-incubation (~5 min) of the cells with C#1 at 10 µM, followed by treatment with a series of acylated ghrelin concentrations ranging from 10^−5^ to 10^−11^ M [20], were assessed. The GHSR antagonist YIL781 was used as a positive control in the beta-arrestin recruitment assay.

### 4.4. LC/MS Analysis of C#1

**Figure 7:**
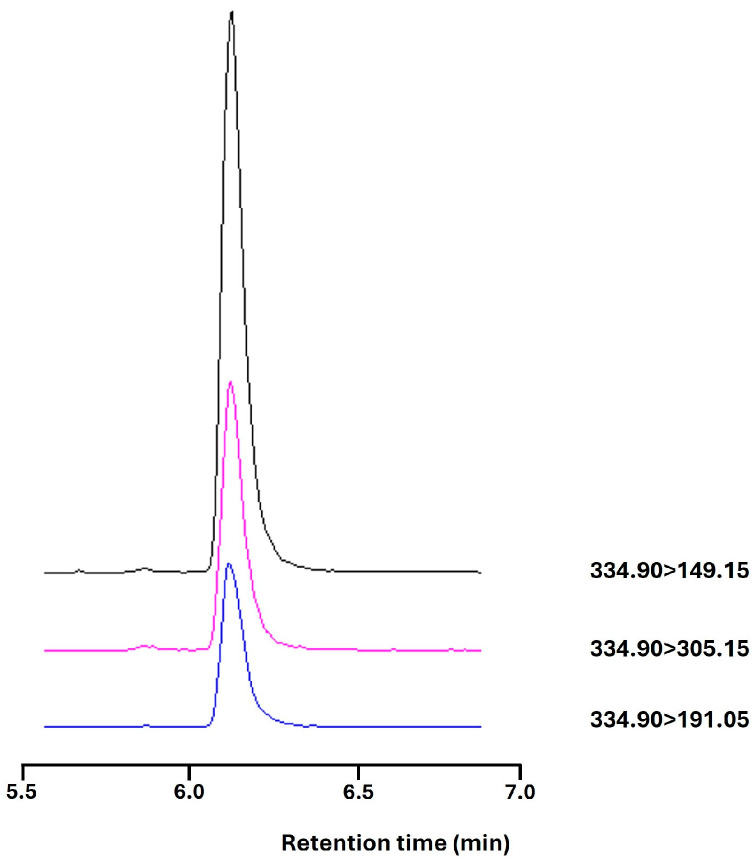
C#1 on LC-MS with an elution time of 6.1 min and a mass transition of C#1 334.9000 > 149.1500(+) (black), >305.1500(+) in purple and >191.0500(+) in blue.

A new assay was developed to measure C#1 in serum samples. Dog EDTA plasma (100 µL) was extracted with 1 mL chloroform (analytical grade, stabilized with 0.5–1.0% ethanol, Merck, Amsterdam, the Netherlands). The mixture was vigorously shaken for 5 s and vortexed for 10 s, and centrifuged at 3000× *g* for 10 min. Next, 0.5 mL of the chloroform layer was evaporated to dryness using a gentle stream of nitrogen. The residue was redissolved in 500 µL methanol (MeOH) (LC-MS grade, Biosolve, Valkenswaard, the Netherlands) with 0.1% formic acid (Biosolve). The standard curve was obtained by adding a standard solution of 100 µM compound C#1 (dissolved in DMSO, Merck) to dog EDTA plasma and diluting it to a concentration range of 3–400 nM. Standards were extracted using the same protocol and were always run in the same run as the samples to correct extraction efficiency. Chemical analysis was conducted by LC-ESI-MS/MS using a Shimadzu Nexera Series LC system coupled with a Shimadzu 8050 triple quadrupole MS operating in the MRM mode. One µL of the sample was injected into a Phenomenex Luna RP-C18(2) column (150 × 2 mm, 5 µm pore size). The mobile phase consisted of MQ water and methanol (MeOH), both of which had 0.1% formic acid. The gradient program consisted of 10% MeOH (0–1 min), 90% MeOH (4–6.9 min), and 10% MeOH (7–10 min) with a flow rate of 0.2 mL/min. The column temperature was maintained at 40 °C. The eluate was diverted to waste until 5.5 min (Figure 7). After elution, the compound was ionized using the positive ion ESI mode. The interface temperature was 300 °C, the heat block temperature was 400 °C, and the DL temperature was 250 °C. Nebulizing gas was set at 3 L/min. The precursor–product mass transition settings were 334.9–149.15 for quantification and 334.9–305.15 for qualification purposes.

### 4.5. Statistical Analysis

Data were analyzed using GraphPad Prism (version: 10.0.2.232) for statistical evaluation and graphical presentation. Depicted are the mean ± SD together with individual data points. The first normal distribution was tested. In the case of normal distribution, repeated measures of one-way ANOVA were used with a Tukey post-hoc correction for multiple testing. Not normally distributed data were evaluated with a non-parametric test (Friedman) and corrected with Dunn’s test. *p* < 0.05 was considered significant. Significance: * *p* < 0.05, ** *p* < 0.01, *** *p* < 0.005 and **** *p* < 0.001.

## 5. Conclusions

The current trial shows a decrease in plasma IGF-1 after treatment of two cohorts of dogs with C#1; however, a concomitant increase in plasma GH was expected. Instead, plasma GH concentrations also decreased. Therefore, the current dog study cannot prove GHR inhibition by C#1. Higher dosing is only possible when treatment periods are shorter due to the adverse toxic effects of C#1. Nevertheless, since the intracellular inhibition of GHR signaling as an anticancer treatment using small molecules remains attractive, C#1 may be used as a lead molecule to develop similar compounds with a better effectivity/toxicity ratio. Further research is also necessary on the potential inhibition of the acylation of ghrelin by C#1 to explain the decreased plasma GH concentrations. Finally, C#1 has also been identified in vitro as a DHFR inhibitor and needs to be tested to determine whether it can overcome methotrexate resistance in tumor models.

## Figures and Tables

**Table 1:** Plasma clinical chemistry during 5 weeks of administration of C#1 at an oral dose of 0.1 mg/kg daily. Given are the mean ± SE. * = *p* < 0.01 different from week 0.

	Week 0	Week 2	Week 3	Week 5
Urea (mmol/L)	5.4 ± 0.5	6.9 ± 0.4 *	6.6 ± 0.3	5.4 ± 0.6
Creatinine (µmol/L)	56 ± 4	59 ± 3	56 ± 3	55 ± 3
Glucose (mmol/L)	5.9 ± 0.2	5.9 ± 0.2	5.5 ± 0.2	5.9 ± 0.2
AF (U/L)	151 ± 48	139 ± 36	128 ± 30	108 ± 23
ALAT (U/L)	57 ± 11	56 ± 11	59 ± 13	52 ± 11
ASAT (U/L)	28 ± 2	27 ± 1	27 ± 1	26 ± 2
Bile acids (µmol/L)	16 ± 7	30 ± 18	23 ± 12	35 ± 27
Total bilirubin (µmol/L)	3.4 ± 0.2	3.2 ± 0.3	3.3 ± 0.2	3.0 ± 0.3 *
Triglycerides (mmol/L)	1.4 ± 0.2	1.6 ± 0.2	1.8 ± 0.2	0.9 ± 0.1
Folic acid (nmol/L)	22 ± 2	33 ± 3 *	30 ± 2 *	16 ± 2
Vitamin B12 (pmol/L)	455 ± 39	375 ± 29	387 ± 32	383 ± 32
TLI (µg/L)	30 ± 4	36 ± 3 *	37 ± 3 *	30 ± 3

**Table 2:** Plasma complete blood count (CBC) during 5 weeks of administration of C#1 at an oral dose of 0.1 mg/kg daily. Given are the mean ± SE.

	Week 0	Week 2	Week 3	Week 5
Hemoglobin (mmol/L)	9.9 ± 0.5	9.5 ± 0.4	10.1 ± 0.5	10.0 ± 0.4
Hematocrit (L/L)	0.46 ± 0.02	0.45 ± 0.02	0.48 ± 0.02	0.47 ± 0.02
MCV (fl)	68.0 ± 1.5	68.1 ± 1.3	69.1 ± 1.3	69.2 ± 1.4
MCH (fmol)	1.44 ± 0.03	1.45 ± 0.03	1.46 ± 0.03	1.48 ± 0.04
RDW (%)	14.3 ± 0.6	14.6 ± 0.6	15.1 ± 0.6	15.3 ± 0.6
Reticulocytes (×10^9^/L)	0.66 ± 0.12	0.84 ± 0.16	1.05 ± 0.22	0.88 ± 0.18
Leucocytes (×10^9^/L)	8.2 ± 0.6	8.0 ± 0.4	8.4 ± 0.5	8.3 ± 0.7
Lymphocytes (×10^9^/L)	2.2 ± 0.3	2.0 ± 0.1	2.3 ± 0.2	2.1 ± 0.3
Monocytes (×10^9^/L)	0.37 ± 0.03	0.40 ± 0.03	0.37 ± 0.02	0.33 ± 0.03
Segments (×10^9^/L)	5.3 ± 0.5	5.3 ± 0.3	5.4 ± 0.3	5.5 ± 0.6
Eosinophils (×10^9^/L)	0.41 ± 0.06	0.36 ± 0.07	0.37 ± 0.07	0.33 ± 0.07
Thrombocytes (×10^9^/L)	412 ± 49	415 ± 56	456 ± 59	423 ± 54

**Table 3:** Main pathologic findings in 6 dogs treated for prolonged periods with oral administration of C#1. Dogs A-D were treated for 90 days with C#1. Dogs E and F were taken out of the study after 42 days and treated with the vehicle only until day 90.

Dog	Main Conclusion
A	Mammary carcinoma (grade 2); lipoma Mild glomerulopathy and interstitial nephritis
B	Follicular cell carcinoma thyroid Benign mixed mammary tumor
C	Follicular cell carcinoma thyroid Nephritis
D	Suspected large cell lymphoma kidney
E	Moderate lymphoplasmacytic enteritis Mild membranous glomerulopathy
F	Slight gastritis Mild enteritis

## Data Availability

Data is contained within the article or Supplementary Materials.

## Supplementary Materials

The following supporting information can be downloaded at: https://www.mdpi.com/article/10.3390/ph17101381/s1, Table S1 and Figure S1: Summary of CYP inhibition by C#1 (BM001) in human liver microsomes; Table S2: Pharmacokinetics of compound C#1 in mice.
