# Supplementary material for: In Vivo Effects of a GHR Synthesis Inhibitor During Prolonged Treatment in Dogs
Source: Pharmaceuticals (Basel). 2024 Oct 16;17(10):1381. doi: 10.3390/ph17101381 (PMC11510805; doi:10.3390/ph17101381)
Supplement: Supplementary file 1 [file pharmaceuticals-17-01381-s001.zip › pharmaceuticals-3203460-supplementary.pdf]

Supplemented data:

Table and Figure S1: Summary of CYP inhibition by C#1 (BM001) in human liver microsomes

CYP450	C#1 (BM001)		Reference Inhibitor		
	IC_{50} (μM)	Maximum Inhibition (%)	Inhibitor	IC_{50} (μM)	I_{max} (%)
CYP3A4 (Midazolam)	>10 μM	74% at 20 μM	Ketoconazole	0.037	100
CYP3A4 (Testosterone)	>10 μM	34.5% at 20 μM	Ketoconazole	0.04	100
CYP2D6	>10 μM	59% at 20 μM	Quinidine	0.04	94
CYP2C9	>10 μM	No inhibition	Sulphaphenazole	0.16	98
CYP2C8	>10 μM	No inhibition	Quercetin	3.3	88
CYP2C19	>10 μM	No inhibition	Nootkatone	69.2	87
CYP1A2	>10 μM	No inhibition	Furafylline	0.40	94
CYP2B6	>10 μM	No inhibition	Ticlopidine	0.03	99

It is concluded that C#1 (BM001) did not show significant inhibition of human CYP450 3A4, 2D6, 2C9, 2C8, 2C19, 1A2 and 2B6 (IC_{50} > 10 μM)

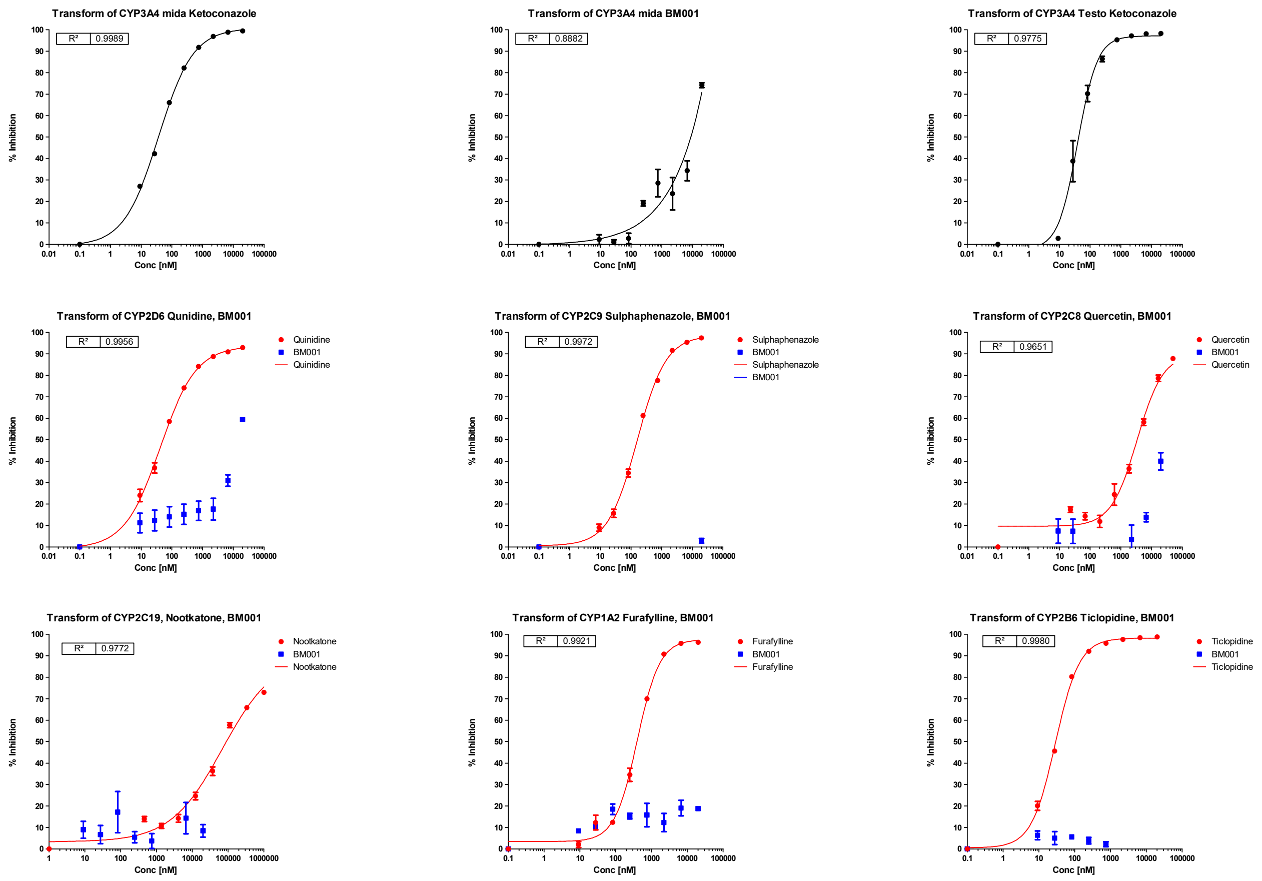

Table S2: Pharmacokinetics of compound C#1 in mice

Parameter	Estimate		
Dose	5 mg/kg	10 mg/kg	15 mg/kg
Ke (1/h)	0.028	0.010	0.027
T1/2 (h)	24.4	67.2	26.1
Tmax (h)	4	8	4
Cmax (ng/ml)	274	550	879
Tlast (h)	24	24	24
Clast (ng/ml)	115	373	520
AUClast (h*ng/ml)	4147	10807	16344
AUCINF (h*ng/ml)	8186	46982	35898
AUC_%Extrap	49	77	54
V/F (ml/kg)	21511	20633	15725
Cl/F (ml/h/kg)	611	213	418
